# Response to Khoodoruth et al., “Atypical presentation of TRPV3 variant: Cerebral palsy and intellectual disability without dermatologic features of Olmsted syndrome”

**DOI:** 10.1016/j.jdcr.2024.12.013

**Published:** 2024-12-24

**Authors:** Travis Frantz, David Kirwin, Angela Crotty, Willis Lyford

**Affiliations:** Department of Dermatology, Naval Medicine Readiness and Training Command San Diego, San Diego, California

*To the Editor:* We read with interest Khoodoruth et al’s^1^ case report of a patient with cerebral palsy and intellectual disability who was identified as having a variant of the transient receptor potential vanilloid 3 (TRPV3) gene but did not have any cutaneous stigmata of Olmsted syndrome (OS).

There is paucity of literature, despite numerous case reports, that associates OS with intellectual disability.^2^ The initial description by Olmsted^3^ and a subsequent case reported by Atherton et al^4^ describes grossly delayed psychomotor skills in young patients with OS. Palmoplantar keratoderma of OS typically begins to develop within the first year of life, which undoubtedly has a profound effect on motor and social development and thus may be confounding. Atherton et al^4^ opine in their report that the perceived cause of the patient’s delay was due to emotional deprivation and inability to develop manual skills. Thus far, there is little evidence to support intellectual disability as clinical feature of OS.

## Conflicts of interest

None disclosed.
